# Diabetic retinopathy: could the alpha-1 antitrypsin be a therapeutic option?

**DOI:** 10.1186/0717-6287-47-58

**Published:** 2014-11-18

**Authors:** Gustavo Ortiz, Juan P Salica, Eduardo H Chuluyan, Juan E Gallo

**Affiliations:** Nanomedicine and Vision Group, Facultad de Ciencias Biomédicas, Universidad Austral, Buenos Aires Pilar, Argentina; Departamento de Farmacología,Ciudad Autónoma de Buenos Aires, Universidad de Buenos Aires, Buenos Aires, Argentina; Ciudad Autónoma de Buenos Aires, CONICET (Consejo Nacional de Investigaciones Científicas y Técnicas), Buenos Aires, Argentina

**Keywords:** Diabetic retinopathy, Alpha-1-antitrypsin, Diabetes, Endogenous anti-inflammatory agents, Retinal inflammation, NF-kB

## Abstract

Diabetic retinopathy is one of the most important causes of blindness. The underlying mechanisms of this disease include inflammatory changes and remodeling processes of the extracellular-matrix (ECM) leading to pericyte and vascular endothelial cell damage that affects the retinal circulation. In turn, this causes hypoxia leading to release of vascular endothelial growth factor (VEGF) to induce the angiogenesis process. Alpha-1 antitrypsin (AAT) is the most important circulating inhibitor of serine proteases (SERPIN). Its targets include elastase, plasmin, thrombin, trypsin, chymotrypsin, proteinase 3 (PR-3) and plasminogen activator (PAI). AAT modulates the effect of protease-activated receptors (PARs) during inflammatory responses. Plasma levels of AAT can increase 4-fold during acute inflammation then is so-called acute phase protein (APPs). Individuals with low serum levels of AAT could develop disease in lung, liver and pancreas. AAT is involved in extracellular matrix remodeling and inflammation, particularly migration and chemotaxis of neutrophils. It can also suppress nitric oxide (NO) by nitric oxide sintase (NOS) inhibition. AAT binds their targets in an irreversible way resulting in product degradation. The aim of this review is to focus on the points of contact between multiple factors involved in diabetic retinopathy and AAT resembling pleiotropic effects that might be beneficial.

**Electronic supplementary material:**

The online version of this article (doi:10.1186/0717-6287-47-58) contains supplementary material, which is available to authorized users.

## Introduction

The overall prevalence of diabetic retinopathy (DR) in diabetic patients is about 34% worldwide and it is the leading cause of blindness in the working population (16–64 years old) [1]. The underlying mechanisms of this disease include degenerative and inflammatory changes as well as remodeling processes of the extracellular-matrix (ECM) leading to pericyte and vascular endothelial cell damage that severely affects the retinal microcirculation. In turn, this causes hypoxia, vascular endothelial growth factor (VEGF) release and angiogenesis [2–5]. Neovessels grow in the retina and also into the vitreous, and could induce hemorrhages due to their fragile walls [6, 7]. In advanced stages the development of vitreoretinal fibrosis promotes retinal traction and detachment [8]. It has widely been demonstrated that this process is one of the previous steps to blindness.

Unfortunately, the ophthalmic therapy for diabetic retinopathy is focused on severe stages of the disease. The treatment is carried out when it reaches the so-called pre-proliferative stage using pan-retinal photocoagulation; development of macular edema is treated with focal photocoagulation and anti-VEGF agents; presence of retinal detachment requires vitreoretinal surgery [9]. The development of molecules to treat diabetic retinopathy in early stages is scarcely explored. New insights into pharmaceutical molecules and the recent advances in regenerative medicine should be exploited in order to find a treatment for early DR.

## Review

### - AAT and inflammation

#### Protease-activated receptors

It is well known how alpha*-* 1 anti-trypsin (AAT) binds and inhibits serum serine proteases such as elastase, trypsin, thrombin and proteinase-3 (PR-3) [10]. These serin proteases are considered key mediators of the innate immune response [11, 12] and can activate specific receptors named protease-activated receptors (PARs) on the membrane of immune cells such as neutrophils, eosinophils and macrophages. PARs are a family of four receptors (PAR1-4) involved in the intracellular signaling cascade and PAR-1 and PAR-4 appear to be essential during inflammatory responses [13]. In neutrophils, cell activation is accompanied by Akt (also known as protein kinase B) phosphorylation, rise of intracellular Ca^+2^ and formation of actin filaments, leading to better cell motility [14]. The crucial role of PARs activation during disease progression was revealed in animal models of inflammation such as gastrointestinal diseases, neuroinflammatory and neurodegenerative processes, skin, or allergic responses [11] and insulin-deficient murine type 1 diabetes models [12]. Moreover, the expression of mRNA of the four members of PARs was found in the postnatal eye and in the retina of adult rat [15]. PAR-2 is expressed in a variety of cells, including neuronal tissue, leukocytes, and vascular endothelial cells [16] and it was found involved in neovascularization processes of proliferative retinopathies [17]. Furthermore, PAR-2 has a link between pro-inflammatory and pro-angiogenic effects mediated by TNF-α, via MEK/EK1/2 pathway in the retina [17]. In summary, the inhibition of serine proteases that activate PARs could contribute to decreasing the inflammatory and pro-angiogenic process.

### Reactive oxygen and nitrogen species

It is known that reactive oxygen species (ROS) are generated during diabetic retinopathy [18, 19]. Particularly, superoxide anion production by polymorphonuclear cells (PMNs), was found to be higher in patients with DR than in patients without DR, suggesting that ROS may have a role in retinopathy development [20]. In eosinophils, a target of AAT, trypsin was able to induce superoxide anion production via PAR-2 [21]. Also reactive nitrogen species (RNS) such as nitric oxide (NO) could be modulated by AAT [22]. Du et al., observed a significant increase in superoxide, NO, cyclooxygenase (COX)-2 and leukostasis within retinal microvessels in a model of streptozotocin-treated diabetic rats. These effects were suppressed using a p38 mitogen-activated protein kinase (MAPK) inhibitor [23]. However, the role of AAT in the activation of p38 and ERK1/2 MAPK could not be demonstrated in *in vitro* studies of murine RAW 264.7 macrophagic cells stimulated with combined LPS and IFN-γ [23]. Therefore the relationship between AAT and superoxide anion production of NO seems to be partly regulated via MAPK in diabetic retinal microvessels, but not in cells of the innate immune system such as macrophages. However, some evidences suggest that the development of retinal neovessels requires the involvement of macrophages [24, 25]. The number of macrophages rises in the vitreous and in the retina of animals with oxygen induced retinopathy [26]. Also, a mutation of macrophage colony stimulator factor was reported to reduce retinal neovascularization [27]. These findings support the hypothesis that the activation and migration of macrophages contribute to the pathogenesis of retinal neovascularization.

### Neutrophil chemotaxis

In the absence of any exogenous stimuli, AAT inactivates calcium-dependent cysteine protease calpain I (μ-calpain) and concomitantly induces random neutrophil migration and polarization. Moreover, rho GTPases are rapidly activated, and neutrophils show increase phosphorylation of ERK 1/2. Also, AAT inhibits neutrophil adhesion to fibrinogen [28]. Bergin et al. [14] have provided evidence that AAT modulates neutrophil chemotaxis by association with neutrophil membrane lipid rafts, interacting with the glycosylphosphatidylinositol linked (GPI-linked) membrane protein FcγRIIIb and inhibiting ADAM- 17 activity, a tumor necrosis factor alpha converting enzyme. Neutrophil migration is a process that occur due to chemotaxis [29], an event that is present in diabetic retinopathy [30].

**Figure 1:**
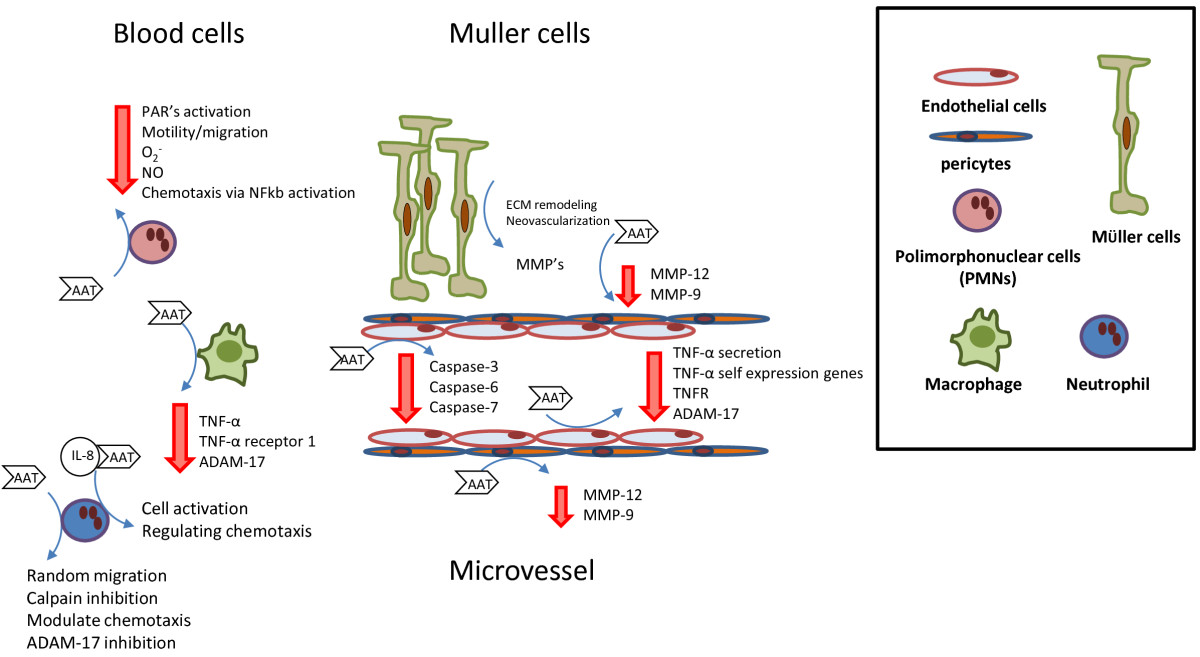
**The interaction of AAT with Blood Cells and Müller Cells might influence the development of diabetic retinopathy.**

On the other hand, glycosylated AAT can bind to IL-8, a ligand for CXCR1 (chemokine receptor 1), and the AAT-IL-8 complex formation can prevent IL-8 interaction with CXCR1 regulating neutrophil chemotaxis [14]. In response to IL-8, the cell is activated resulting in actin filament formation and cytoskeletal rearrangement, via Akt (also known as PKB) phosphorylation and Ca ^2+^flux. Thus, when IL-8 binds to AAT it cannot interact with CXCR1 and cell activation is inhibited (Figure 1).

### CD40 and NFkB

It has been observed that CD154 (CD40 ligand) plays a key role in the production of pro-inflammatory cytokines and it has been linked to various autoimmune diseases with microvascular complications, like diabetes mellitus [31–33]. In vitro studies using Jurkat E6.1 T-cells demonstrated that the soluble form of CD154 (sCD154) is released from T-cells by ADAM10 and ADAM17 upon CD40 ligation [34]. Interestingly, a recent investigation performed in CD40 knock-out mice showed that these animals exhibited diminished inflammatory responses and they were protected from the development of diabetic retinopathy, suggesting that CD40 promotes the development of early diabetic retinopathy [35].

It was observed that AAT was able to inhibit nuclear transcriptional factor-kB (NF-kB) activation in a variety of animal models preventing PMN chemotaxis and the development of acute inflammation [36–38]. Activation of NF-kB induced by diabetes and high glucose regulates a pro-apoptotic program in retinal pericytes [39] and is well known that these cells are affected early in diabetic retinopathy [40].

### Tumor necrosis factor-alpha and leucocytes

The effect of AAT on tumor necrosis factor alpha (TNF-α) was demonstrated in a microarray study in human endothelial lung cells. The co-administration of AAT inhibited 25% of genes up-regulated by TNF-α including TNF-α-induced self-expression. These effects were equally achieved when oxidized AAT, a modified form of AAT, lacking serine protease inhibitor activity was used [41]. AAT inhibited TNF-α receptor-1 up-regulation and significantly reduced TNF-α secretion. These results were associated with inhibition of TNF-α-converting enzyme activity or ADAM17. Furthermore, AAT inhibited calpain activity, whose activation by TNF-α contributed to decreasing intracellular AAT concentrations. All these data indicate that AAT initially facilitates acute responses of the endothelium to TNF-α, followed by selective inhibition of TNF-α-induced-self amplification, which may assist the vasculature in the resolution of chronic inflammation [42].

Intermittent infusions of alpha 1-antitrypsin were shown to be beneficial in the treatment of patients with alpha 1-antitrypsin deficiency [43] and augmentation therapy caused decreased neutrophil infiltration [44, 45]. Leukocytes and proteins that govern leukocyte adhesion to endothelial cells play a causal role in retinal abnormalities characteristic of the early stages of diabetic retinopathy, including diabetes-induced degeneration of retinal capillaries [46, 47]. These facts suggest a possible beneficial use of AAT in early stages of DR.

### Protective effect on beta pancreatic cells

Non-functional circulating AAT (probably due to excessive non enzymatic glycation) was described in type 1 diabetes [48–53]. Additionally, levels of AAT in non-obese diabetic mice (NOD) were found to be half of those seen in the wild type strains [52]. These facts led to the development of gene therapy strategies using recombinant adeno-associated virus-(AAV) carrying murine AAT genes. As a result, AAV-AAT prevents type I diabetes in NOD mice [54]. Alpha-1-Antitrypsin (AAT) has been shown to reduce pro-inflammatory markers and protect pancreatic islets from autoimmune responses in pre-clinical studies [55]. Currently, clinical trials using recombinant AAT are being conducted in type 1 diabetic patients (Table 1). Preliminary results of one study showed better metabolic control probably through a protect effect on beta pancreatic cells that lead to a halt in disease progression. Diabetic retinopathy and other complications would benefit from this systemic therapy. In addition, visual scientists could consider the possibility to develop an ophthalmic treatment of AAT to further prevent or delay diabetic retinopathy.

**Table 1:** **Ongoing clinical trials using AAT in young patients with type 1 diabetes**

NCT	Phase	Age range (years)	Source/dose of AAT (mg)
01304537	II	10 to 25	Glassia®/40-60-80
01319331	I	6 to 45	Aralast NP
01183468	II	8 to 35	Aralast NP

## - AAT role in cell death

Many studies have determined the ability of AAT to inhibit caspases. These are involved in cell death by apoptosis, as inducers or effectors [56]. The role of AAT in caspase-3 inhibition was described in murine lung endothelial cells and in murine pancreatic beta cells [57, 58]. Also, AAT was capable of inhibiting executing caspase-6 and −7 in lung microvascular endothelial cells [56]. Similar results were reported in animal models of diabetic retinopathy and also in diabetic patients. Activation of retinal caspases, particularly caspase-3, lead to apoptosis of endothelial cells and pericytes [59, 60]. The capacity of AAT to inhibit caspases could be exploited in order to protect microvasculature from early damage induced by DR (Figure 1).

## - Potential interaction between AAT and Müller cells

Similary to brain astrocytes, Müller cells could produce factors that induce the formation of tight junctions conferring barrier properties to the retinal vessels [61]. They synthesize or store a number of growth factors with trophic or regulatory functions for various cell types in the retina. These characteristics make an assessment of Müller cell function in diabetes relevant to two well-known features of diabetic retinopathy: vascular leakage and capillary obliteration. Indeed, microvascular cell apoptosis occurs in human and experimental diabetic retinopathy [62], and one of the mechanisms leading to apoptosis is loss of survival signals provided by neighboring cells [63]. On the other hand, Müller cells might release metalloproteases (MMPs) that promote the degradation of extracellular matrix (ECM), along with the evidence that MMPs promote cell migration and proliferation. This strongly suggests that Müller cells play an important role in the control of cell and ECM interactions that, in turn, facilitate the development of retinal neovascularization (Figure 1).

It is noteworthy that Müller cells are currently being used in gene delivery. These cells transfected with plasmids or adeno-associated vectors (AAV) containing different constructions are a useful tool to explore different pathways. The retina is an attractive structure for gene therapy approaches because it is surgically approachable, isolated due to the presence of the blood-retinal barrier (BRB) and immunologically privileged. A study demonstrated that accumulation of hypoxia-inducible factor-1α in Müller cells induces the expression of VEGF, which in turn, promotes increased MMP-2 expression and activity in neighboring endothelial cells (EC). MMP-2 expression was detected in endothelial cells of retinal neovessels from proliferative diabetic retinopathy (PDR) patients, whereas MMP-2 protein levels were elevated in the aqueous humor of PDR patients compared with healthy patients [64]. The stability control of the microvasculature through regulation of the extracelullar matrix (ECM) in the retina is essential to avoid progressive development of the disease. AAT could be involved in the control of ECM because of its ability to inhibit MMP-12 and MMP-9. Furthermore, gene therapy using AAT could be a suitable tool for the inhibition of those changes.

The mRNA and protein levels of the complement receptor C5aR were measured in human Müller cells. C5aR was found constitutively expressed in human Müller cells. Up-regulated C5aR expression in Müller cells was promoted by, prostaglandin E2 and hyperglycemia, either individually or synergistically. Signaling through C5aR on Müller cells up-regulated production of IL-6 and VEGF, which promoted the proliferation of human retinal endothelial cells and increased their permeability [65]. Furthermore, IL-6 seems to be involved in the regulation of AAT since human hepatocyte exposure to IL-6 increased the expression levels of AAT [66]. A recent investigation also found increased IL-6 levels in diabetic animals [67]. This information suggests that complement plays a role in disease progression but how this could modulate the activity of AAT and the relationship between AAT and C5aR remains to be verified. However, the use of silencing strategies to reduce the availability of the receptor C5aR in the retina might be beneficial. Similar strategies have already been used in retinal Müller cells [68].

## - AAT and extracellular matrix remodelling

MMPs are a family of enzymes capable of degrading essentially all ECM components [69]. The two major matrix degrading enzymes, known as MMP-2 and MMP-9 were found in the vitreous of eyes with proliferative DR [70]. The main source of these MMPs in vivo may be retinal pigment epithelial cells [71–73]. In the retina of diabetic rats the activation of cytosolic MMP-9 and MMP-2 is an early event, which is followed by their accumulation in the mitochondria [74]. In humans, it was found a positive correlation between vitreous levels of MMP-9 and VEGF with proliferative DR [75], and levels of AAT were found increased in different types of vitreoretinal diseases [76]. Besides, another study found higher vitreous levels of AAT in proliferative DR compared with vitreous levels seen in cases without diabetes mellitus [77]. Another MMP, MMP-12 is mainly produced by macrophages and called both metallo-elastase or macrophage-elastase [78]. An important factor in the development of vascular wall alterations is the degradation of the elastic fiber major protein-elastin [79]. It should be noted that hyperglycemia may directly disrupt elastin formation [80]. In diseases such as chronic obstructive pulmonary disease (COPD), it has been shown that AAT is capable of inhibiting the action of MMP-12. Besides, preliminary results on streptozotocin induced diabetes in rats intravitreally treated with human alpha-1 proteinase inhibitor Prolastin® have shown a higher expression of MMP-12 compared with controls (Ortiz et al. unpublished data). AAT also inhibited MMP-9 in a mouse model of the autoimmune disease bullous pemphigoid [81]. MMP-9 is an important IL-1 inducible protease that is suspected of contributing to the progression of various diseases such as cardiovascular disease, rheumatoid arthritis, COPD and multiple sclerosis [81, 82]. These evidences together suggest that progression of angiogenesis is associated with MMP’s and also with inflammation process in the vitreoretinal diseases. It is important to better understand these processes, to avoid the progression of the disease.

Recent studies on the role of epigenetic patterns in streptozotocin-induced diabetic rats reported an altered pattern of methylation of histone H3K4 H3K9 located in the promoter of MMP-9. The activity of Lysine-specific demethylase 1 (LSD1) was found elevated by 50% and gene and protein expression was 2-fold augmented. Gene activation markers, acetyl H3K9 and NF-kB (p65 subunit) recruitment were found to be increased by about 18-fold and 30-fold, respectively [83]. Epigenetic changes modify the expression pattern of MMP’s occurring at early stages in the development of DR. To ameliorate these changes the use of molecules that neutralize MMP’s action seems to be necessary.

The outgrowth of mouse retinal ganglion cells (RGCs) is co-regulated by MMP-2 and another membrane type 1 MMP (MT1-MMP) [84]. Furthermore, in an ex vivo retinal explant model MMPs were shown to be beneficial factors in axonal regeneration. On the other hand, CD44 proteolysis in T-cells is involved in migration and function of self-reactive T-cells, and a study using three MMP inhibitors in NOD mice found that MT1-MMP has a unique involvement in type 1 diabetes development [85].

## - Vessel walls and capillaries might be protected by AAT

Pericyte loss and microaneurysm formation are hallmarks of early changes in the retinas of diabetic patients [86]. After induction of diabetes in rodents, reduction of pericyte number in retinal capillaries is the earliest morphological change, followed by the formation of increased number of acellular-occluded capillaries, occasional microaneurysms, and thickening of the vascular basement membrane [87]. With progressive vascular occlusions in the human diabetic eye, the retina responds with either a progressive increase of vascular permeability leading to retinal edema, or the formation of new vessels that finally proliferate into the vitreous [5].

Pericytes can control endothelial cell proliferation and angiogenesis, both under physiological and pathological conditions [88–94]. DR is morphologically characterized by pathological changes in the retinal capillaries. The primary and predominant characteristics are the loss of pericytes and the progressive occlusion of capillaries [3, 86]. Several research groups [39, 95, 96] have reported that cultured retinal pericytes exposed to high levels of glucose (25–30 mmol/l) for 7 days or more show a higher rate of apoptosis than cells grown at 5.5 mmol/l glucose. Besides, it has been found that retinal pericytes play a key role in the stabilization of endothelial cells protecting them from hypoxic insults and angiogenic stimuli [4].

Other research groups working on animals at 10 months post diabetes-induction have reported significant increases in the number of degenerate (acellular) capillaries and pericyte ghosts compared to non-diabetic animals. However, when the inhibitor of p38 MAPK was used, all these abnormalities were significantly diminished [23].

It is known that bone-marrow-stem-cells (BMSCs) appear to act primarily through their incorporation into the retina as endothelial cells, microglia, and photoreceptors [97–101]. Also, pericytes can be derived from BMSCs [102], but this does not appear to be a predominant differentiation pathway for these cells when injected into the eye [98, 103]. A recent study showed that pericytes obtained from adipose-derived stem cells (ASCs) protect against retinal vasculopathy. It is noteworthy that ASCs express pericyte-specific markers in vitro, and when they were intravitreally injected into the eye of a mouse model of oxygen-induced-retinopaty (OIR) they were capable of migrating and integrating in the vasculature [104].

The breakdown of the inner blood-retinal barrier (iBRB) is also a feature of experimental diabetes in animal models, being observed as early as 1-2-weeks post-diabetes induction in rodents [105, 106]. It is well established that this lesion occurs early in clinical diabetic retinopathy [107].

Advanced-glication-end products (AGEs) are known to induce expression of the potent angiogenic agent VEGF in the retina in vivo [108, 109] and in retinal cells in vitro [110, 111]. It has been demonstrated that in short-term diabetic rodents (3 weeks post induction of streptozotocin 165 mg/kg) inhibition of AGEs prevents disruption of iBRB [112]. Besides, AGEs mediated expression and secretion of TNF-α in rat retinal microglia [113].

**Figure 2:**
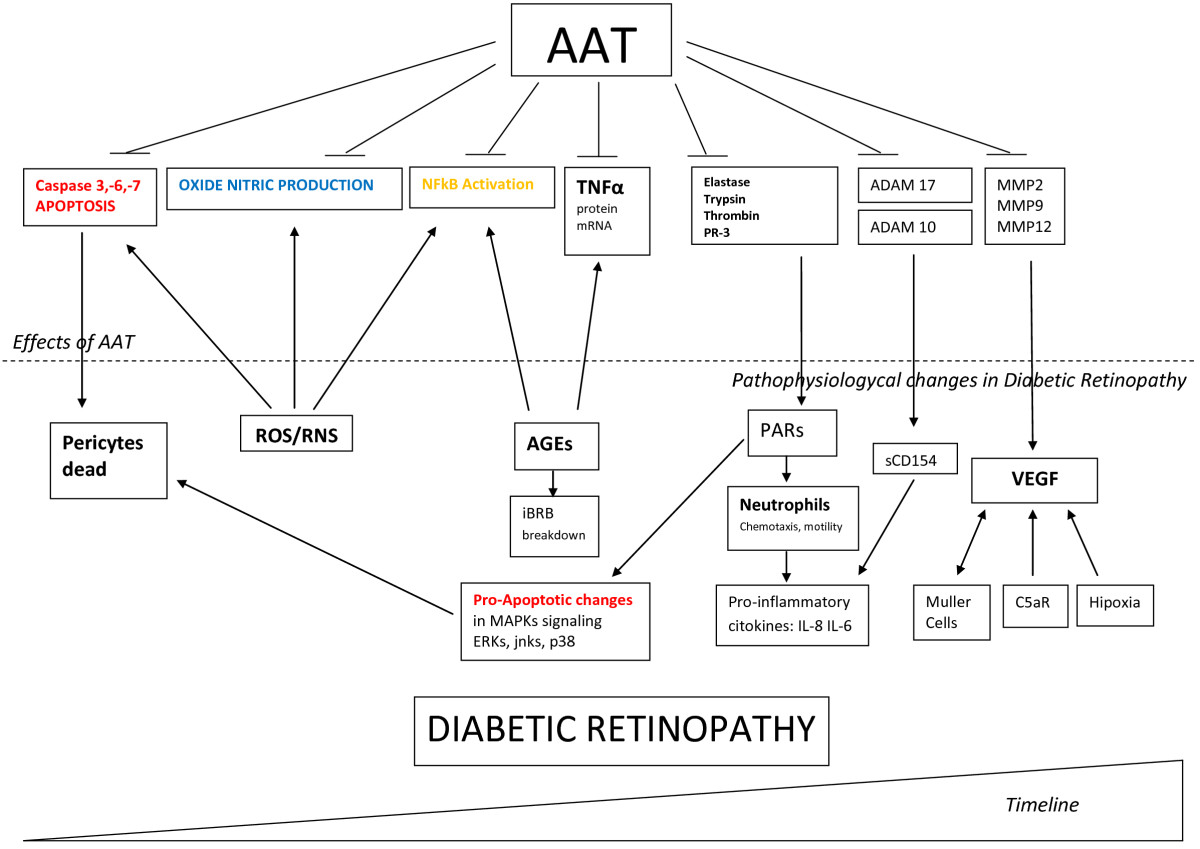
**AAT might ameliorate DR progression inhibiting many key pathways of inflammation in early and advanced disease.** AAT could inhibit several pathophysiological changes that occur during DR. In early stages AAT can inhibit effector caspases preventing the loss of pericytes. In turn, the resulting production of NO could be decreased. Both ROS and AGEs stimulate production of two proinflammatory key molecules: NFkb and TNF-α. Inactivation of these molecules may be performed partially by AAT. During chronic inflammatory processes AAT can inhibit activation of PARPs by blocking the action of serine proteases such as elastase, trypsin, thrombin and PR-3. Finally the process of neovascularization requires remodeling of the extracellular matrix, thereby inhibition of several MMP through AAT may partly decrease the action of VEGF. AAT: alpha 1 antitrypsin ROS: Reactive Oxygen Species RNS: Reactive Nitrogen Species NFkB: Nuclear Factor kappa beta TNF-α: Tumor Necrosis Factor alpha PR-3: Proteinase 3 AGEs: Advanced Glycation End products iBRB: Blood Retinal Barrier MAPKs: Mitogen-Activated Protein Kinases ERKs: Extracellular signal-regulated Kinases Jnks: c-Jun N-terminal kinases PARs: Protease Activated Receptors IL-6: Interleukin 6 IL-8: Interleukin 8 ADAM17: Metallopeptidase domain 17 ADAM10: Metallopeptidase domain 10 MMP-2: Matrix Metalloprotease 2 MMP-9: Matrix Metalloprotease 9 MMP-12: Matrix Metalloprotease 12 VEGF: Vascular Endothelial Growth Factor C5aR: Complement 5a Receptor.

We previously pointed out the capacity of AAT to inhibit protease-activated receptors, to diminish neutrophil chemotaxis, to hinder NFkB activation, to reduce the effect of TNF-alpha and also to inhibit caspases. Through these mechanisms described above AAT might protect the structures of the vessel walls of retinal capillaries that are damaged in DR development.Figure 2 schematizes the above data regarding the involvement of AAT in different pathways during DR progression.

## Conclusions

The above data support the potential protective role of AAT in diabetic retinopathy as a result of its multiple activities and anti-inflammatory properties. AAT is able to inhibit key pro-inflammatory molecules such as NF-kB and TNF-α, as well as all serine proteases involved in activating PARs. Taking into account that activated PARs control neutrophil chemotaxis and motility, a hallmark of inflammatory chronic processes such as those present in diabetic retinopathy, AAT could be administered in the early or advanced stages of DR for the patients to achieve a therapeutic benefit.

Anti-apoptotic properties inhibiting caspase 3, 6, 7 could be beneficial in the pathogenesis of DR and any neurodegenerative process that may occur. Indirect anti-angiogenic features in the retinal microvasculature could decrease ECM remodeling. Because AAT could delay the damage induced by DR, early use of AAT therapy may be an effective strategy to prevent or hinder the progression of diabetic retinopathy.

## Authors’ original submitted files for images

Below are the links to the authors’ original submitted files for images. Authors’ original file for figure 1 Authors’ original file for figure 2
