# Supplementary figures and images for: Diabetic retinopathy: could the alpha-1 antitrypsin be a therapeutic option?
Source: Biol Res. 2014 Nov 18;47:58. doi: 10.1186/0717-6287-47-58 (PMC4335423; doi:10.1186/0717-6287-47-58)

## Blood cells

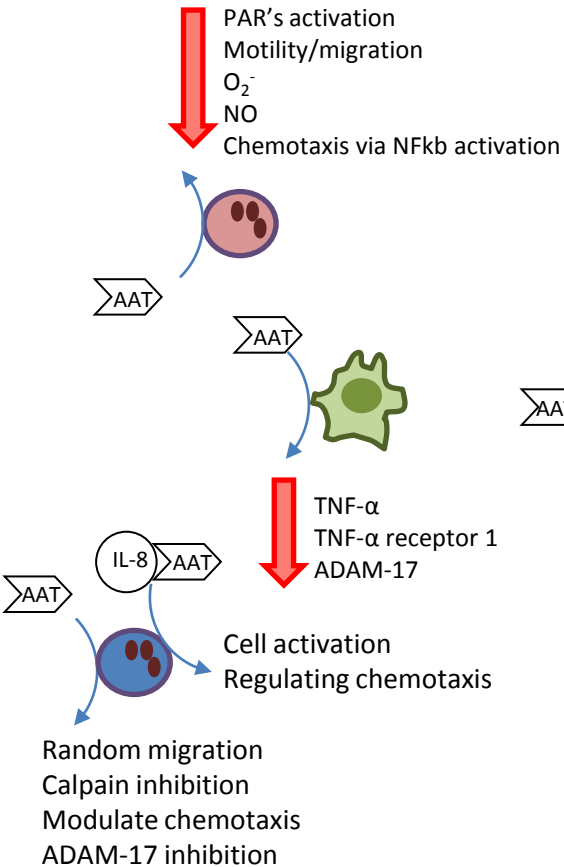

## Muller cells

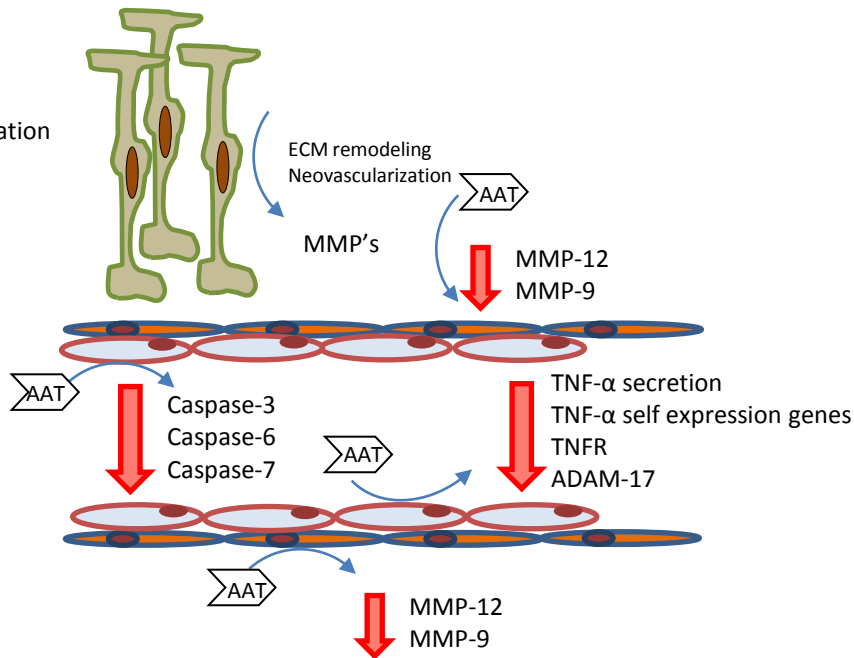

## Microvessel

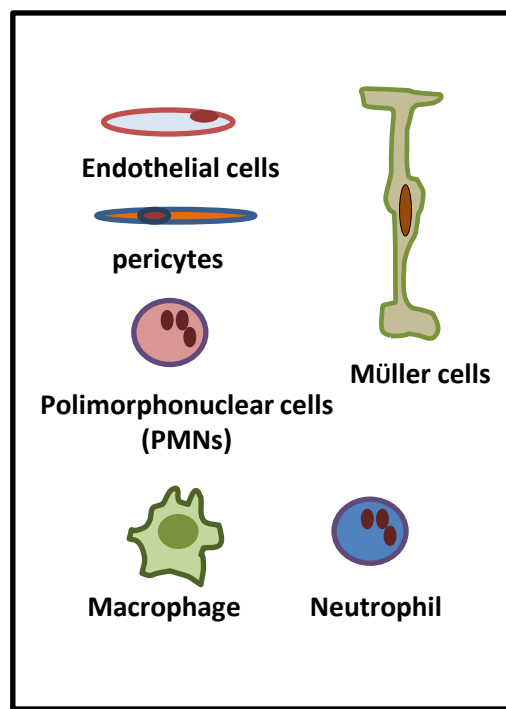

Supplement: Supplementary file 1 — Authors’ original file for figure 1 [file 40659_2014_69_MOESM1_ESM.pdf]

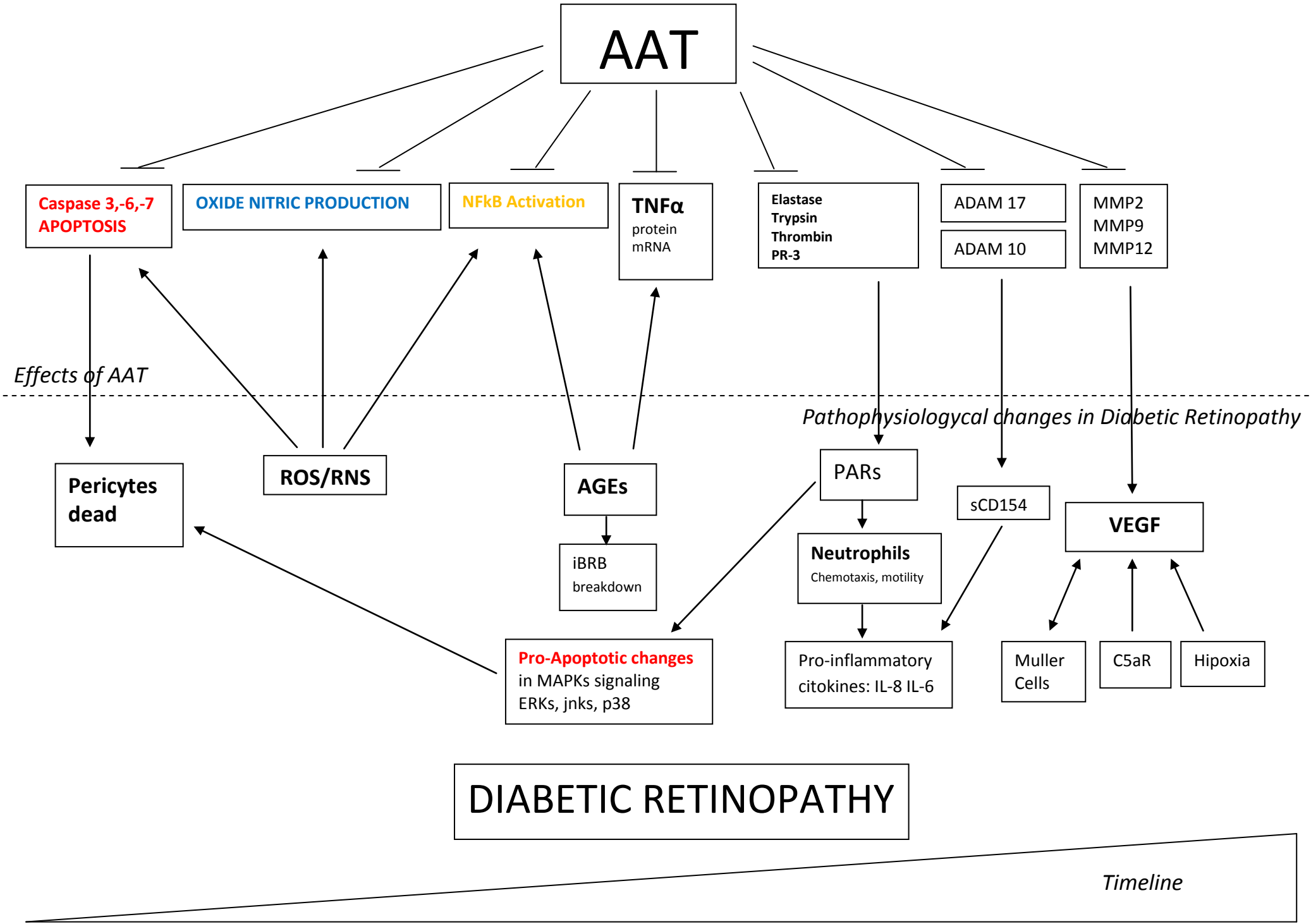

Supplement: Supplementary file 2 — Authors’ original file for figure 2 [file 40659_2014_69_MOESM2_ESM.pdf]
